# Therapeutic benefits of lower limb prostheses: a systematic review

**DOI:** 10.1186/s12984-023-01128-5

**Published:** 2023-01-13

**Authors:** Elke Lathouwers, María Alejandra Díaz, Alexandre Maricot, Bruno Tassignon, Claire Cherelle, Pierre Cherelle, Romain Meeusen, Kevin De Pauw

**Affiliations:** 1 Human Physiology and Sports Physiotherapy Research Group, Vrije Universiteit Brussel, 1050 Brussels, Belgium; 2 Brussels Human Robotics Research Center (BruBotics), Vrije Universiteit Brussel, 1050 Brussels, Belgium; 3 Axiles Bionics, 1130 Brussels, Belgium

**Keywords:** Lower limb, Amputation, Quality of life, Health, Prosthesis, Biomechanics, Physiology, Performance, Psychology

## Abstract

**Background:**

Enhancing the quality of life of people with a lower limb amputation is critical in prosthetic development and rehabilitation. Yet, no overview is available concerning the impact of passive, quasi-passive and active ankle–foot prostheses on quality of life.

**Objective:**

To systematically review the therapeutic benefits of performing daily activities with passive, quasi-passive and active ankle–foot prostheses in people with a lower limb amputation.

**Methods:**

We searched the Pubmed, Web of Science, Scopus and Pedro databases, and backward citations until November 3, 2021. Only English-written randomised controlled trials, cross-sectional, cross-over and cohort studies were included when the population comprised individuals with a unilateral transfemoral or transtibial amputation, wearing passive, quasi-passive or active ankle–foot prostheses. The intervention and outcome measures had to include any aspect of quality of life assessed while performing daily activities. We synthesised the participants’ characteristics, type of prosthesis, intervention, outcome and main results, and conducted risk of bias assessment using the Cochrane risk of bias tool. This study is registered on PROSPERO, number CRD42021290189.

**Results:**

We identified 4281 records and included 34 studies in total. Results indicate that quasi-passive and active prostheses are favoured over passive prostheses based on biomechanical, physiological, performance and subjective measures in the short-term. All studies had a moderate or high risk of bias.

**Conclusion:**

Compared to passive ankle–foot prostheses, quasi-passive and active prostheses significantly enhance the quality of life. Future research should investigate the long-term therapeutic benefits of prosthetics devices.

## Introduction

Lower limb loss is detrimental for physical function and psychosocial health, harming an individual’s quality of life [1, 2]. After lower limb loss, assistive devices are a fundamental part of rehabilitation with the aim to restore daily activities and improve quality of life [3–5]. A prosthesis is generally favoured among assistive devices as it enables one to carry out daily activities as naturally as possible [4, 6, 7]. It additionally helps to enhance self-esteem as it ensures that people diverge as little as possible from the physical appearance of able-bodied individuals [8]. Conversely, prostheses may cause falls and secondary injuries, including low back pain and osteoarthritis of the healthy knee and hip, entailing high medical costs and diminishing quality of life [9–13].

Lower limb prostheses also complicate the performance of daily activities. This performance is affected by several factors, including the type of prosthesis, prosthetic embodiment, the level and cause of the amputation, degree of mobility and presence of comorbidities [14, 15]. These factors complicate tasks such as positioning the foot in space, walking horizontally, going up and down ramps and stairs, crossing obstacles, walking on slippery floors and transitioning between activities [16–18]. For instance, it has been well-established that walking with a lower limb prosthesis results in aberrations in gait kinetic and spatiotemporal parameters compared to able-bodied walking [19]. These aberrations can be attributed to the loss of sensory feedback and the inability of the prosthetic device to mimic the normal muscular function [19, 20]. As a result, bilateral proximal muscle compensations, increased metabolic cost and secondary injuries (e.g. low back pain, arthritis of the sound knee, bilateral hip osteoarthritis, reduced hip bone density of the amputated limb and muscle atrophy) occur [21–26]. Furthermore, people with a lower limb amputation concomitantly show increased structural and functional changes occurring in the brain after an amputation and exhibit a decrease in static and dynamic balance. These unfavourable changes culminate in an increased risk of falling, which leads to reduced quality of life [9, 10, 27, 28]. Besides the physical and biomechanical consequences, the psychosocial impact is equally important and keeps fluctuating throughout the years following amputation [29]. For example, an individual’s functional status has a strong positive influence on overall satisfaction and thus on the quality of life, while the emotional state (i.e., depression and anxiety), body image disturbances, and high pain levels have a negative influence [29, 30]. All these adaptations emphasise the necessity for both short- and long-term research into the effect of ankle–foot prosthetic technological innovations on quality of life.

The current evolution in prosthetic development is shifting from developing passive prostheses to quasi-passive and active prostheses in order to minimise prosthetic related adverse events affecting quality of life [31–36]. This critical aspect of lower limb prosthetic development and rehabilitation might be achieved by restoring the quality of life by increasing mobility, improving psycho-sociological negative implications following amputation, and alleviating gait compensations during daily activities as well as better mimicking able-bodied motions [3, 15, 37, 38].

Therapeutic benefits for people with lower limb amputation might thus arise from technological innovations that could improve their quality of life [31–36]. Nevertheless, quality of life is an inherently intricate concept, and there seems to be no consensus on a single definition constituting quality of life [39]. There is, however, agreement on its multidimensionality and subjectivity, covering health (i.e., physical, social, mental and emotional functioning) and individuals’ perceptions such as pain, relationships and life satisfaction [39, 40]. Differences in quality of life can be investigated through objective (e.g. performance, biomechanical, physiological) and subjective measurements (e.g. psychosocial) in this population [41–43]. Though, no insight is available concerning the impact of passive, quasi-passive and active ankle–foot prostheses on the different dimensions of quality of life. Therefore, the purpose of this study was to systematically review the therapeutic benefits of performing daily activities with passive, quasi-passive and active ankle–foot prostheses in people with a unilateral lower limb amputation.

## Methods

### Search strategy

The review protocol has been registered in Prospero under CRD42021290189. This systematic review has been reported in compliance with the PRISMA 2020 statement, the PRESS guideline and the PERSiST consensus statement [44–46]. A systematic search strategy through four electronic databases (i.e., PubMed, Web of Science, Scopus, and Pedro) was conducted using the PICO acronym (population, intervention, comparison, outcome) on November 3, 2021, followed by a backward reference search. We have not sought and browsed additional data from study registers or other online sources.

### Search string

Table 1 provides the search string used across all databases combining the intended population, intervention, and outcome through the Boolean operator “AND”. The search string has been created by one author (EL) and reviewed by two authors (BT & KDP). We limited the search hits to journal articles written in English published later than 2000. The timeframe was chosen as research into the development and evaluation of lower-limb prostheses only started to advance rapidly in the twenty-first century with the introduction of quasi-passive and active ankle–foot prostheses [47–49].

**Table 1:** Search string

(“Transfemoral amput*” OR “Transtibial amput*” OR “lower limb amput*” OR “limb loss”) **AND** (“quality of life” OR “psych*” OR “biomech*” OR soci*” OR “activities of daily living” OR “mental health” OR “health” OR “physiol*”) **AND** (“active prosth*” OR “bionic” OR “powered prosth*” OR “novel prosth*” OR “passive” OR “microprocess*” OR “quasi-passive” OR “foot” OR “feet” OR “knee*”) Filters: Journal Article, English, from 2000 to 2021	

### Selection criteria

Randomised controlled trials, cross-sectional, cross-over or cohort studies were included. Subjects had to be individuals with a unilateral transfemoral or a transtibial amputation, wearing passive, quasi-passive or active ankle–foot prostheses. We excluded studies on children, and adults with upper limb amputation, bilateral, foot or trans articular knee amputation. The intervention and outcome measures had to include any aspect of quality of life assessed while performing daily activities. Since we aimed at reviewing differences between passive, quasi-passive and active prostheses, we chose to include only articles comparing different ankle–foot prostheses.

### Eligibility assessment

Studies collected through the electronic databases for duplicate removal and eligibility were imported into Rayyan (https://rayyan.qcri.org) [50]. Duplicates were removed using Rayyan’s duplicate identification software, and the remaining duplicates were removed manually. Two authors (EL & MAD) performed a two-stage eligibility assessment. Disagreements were resolved upon consensus and a third author (AM) was contacted if a consensus could not be reached. First, screening was conducted on title and abstract for language, study design, population, intervention, and outcome. Subsequently, the remaining eligible articles were screened on full text following the same criteria.

### Data extraction

The author’s name, year of publication, study design, participants’ characteristics (i.e., number of participants, level of amputation, reason of amputation, gender, age, weight, height, time since amputation), type of prosthesis, intervention, outcome and main results were extracted from the included studies and tabulated by one author (EL).

### Risk of bias assessment

Risk of bias assessment was performed by two reviewers (MAD & AM) using the “The Cochrane 2.0 risk of bias tool” for crossover studies [51]. Disagreement was resolved by consensus between the two author and a third author (EL) if consensus could not be reached. “The Cochrane 2.0 risk of bias tool” consists of eighteen questions to assess the randomization process (n = 5), the deviation from intended interventions (n = 5), the missing outcome data (n = 3), the measurement of the outcome (n = 2) and selection of the reported results (n = 3). Four possible answers could be given to each question: ‘yes’, ‘no’, ‘no information’ and ‘not applicable’. The risk of bias was determined by following the decision tree as indicated in the assessment tool and resulted in a low, moderate or a high risk of bias [51]. Overall low risk of bias across domains was indicated if the study was judged to have a low risk of bias over all of the individual domains. If the study raised some concern in at least one domain but did not present a high risk of bias in any domain, it resulted in an overall moderate risk of bias. If one of the domains had a high risk of bias, by default this resulted in an overall high risk of bias for all domains [51].

## Results

### Study selection

**Fig. 1:**
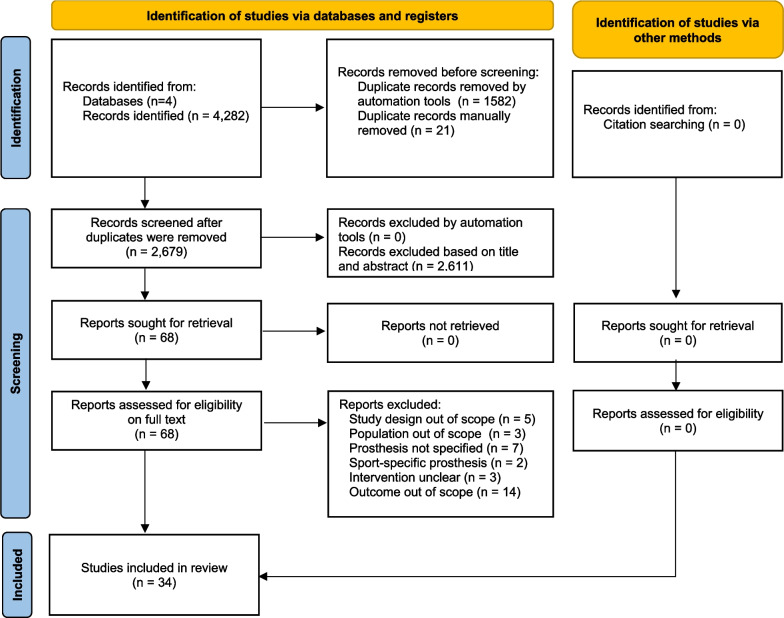
Study selection process

The search yielded 1352 records through PubMed, 1177 through Web of Science, 1656 through Scopus and 97 through Pedro. After screening, 34 studies remained and were included in this systematic review. The results of the selection process are illustrated in Fig. 1.

### Study design and quality assessment

Seventeen studies had a cross-sectional study design [52–68], and 17 had a cross-over design [69–85]. Cross-sectional studies implemented a within-study design. This made them identical to those with a crossover design except for the order in which the devices were evaluated. Therefore, we reported the risk of bias using the same tool for all studies.

**Fig. 2:**
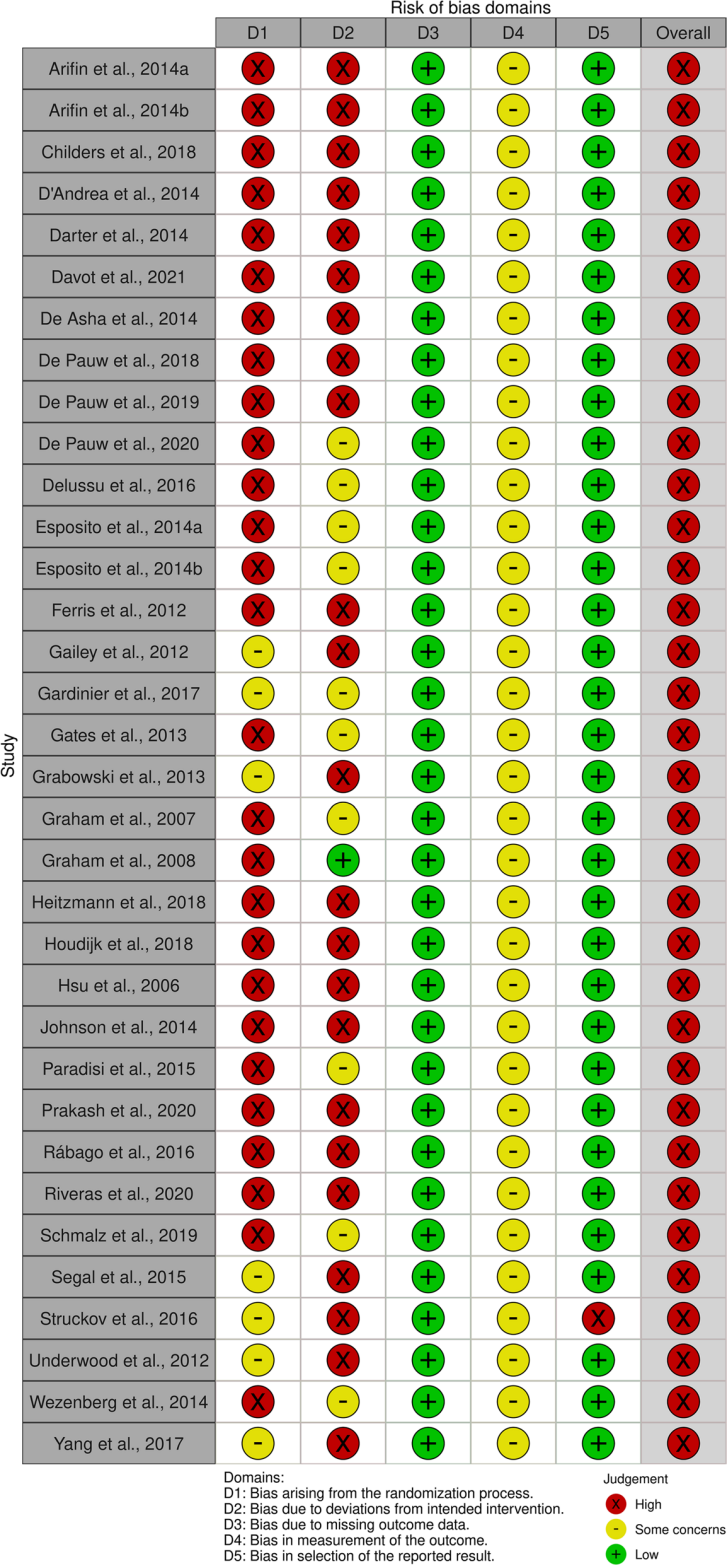
Individual risk of bias per study

**Fig. 3:**
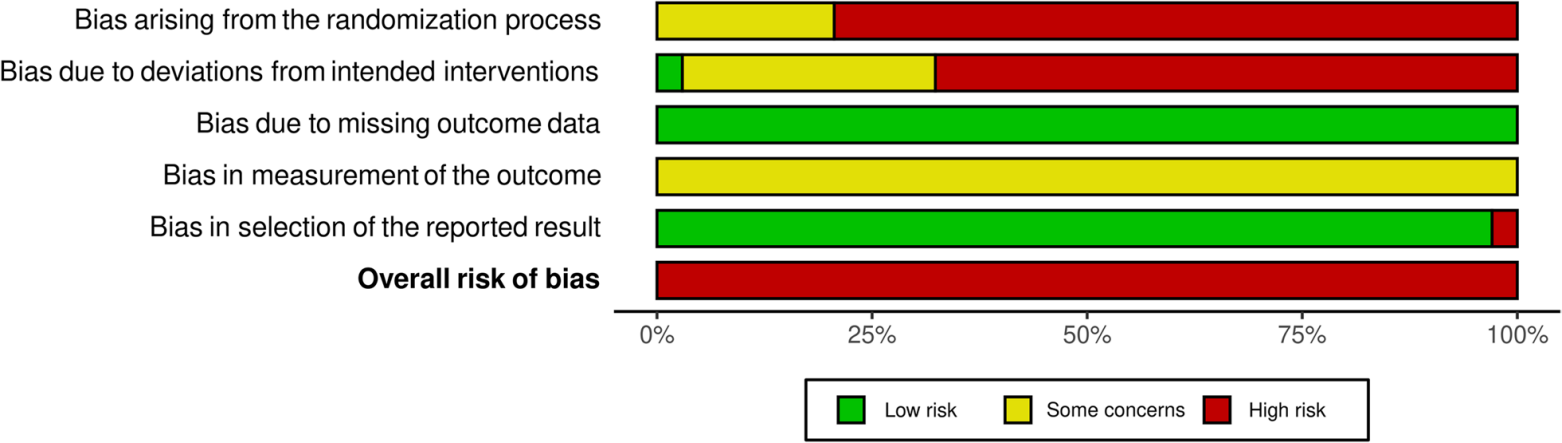
Overall risk of bias

The risk of bias assessment revealed an overall high risk of bias. When we investigated the questions on which the scores were weak, we observed shortcomings in describing the randomization process and reporting deviation from the intended intervention resulting in a high risk of bias. Additionally, we identified minor issues in reporting outcome measurements, entailing a moderate risk of bias. At last, we rated bias due to missing data or reporting a selection of the results as low. Figure 2 details the individual risk of bias per study, and Fig. 3 visualizes the overall risk of bias.

### Study characteristics

Table 2 presents a summary of the study characteristics. Comparison between passive ankle–foot prostheses was most frequently conducted (47%, n = 16) [54, 59–64, 68–71, 73, 80, 81, 84, 85]. Within this category, 14 articles included people with a transtibial amputation, 3 included people with a transfemoral amputation (88% [54, 61–64, 68–71, 73, 80, 81, 84, 85] and 19% [59, 60, 73], respectively), and the mean sample size totalled 13. Ten articles compared quasi-passive with passive prostheses (29%, n = 10) [53, 66, 67, 72, 74–76, 82, 83, 86]. All articles included people with a transtibial amputation (100%, n = 10 [53, 66, 67, 72, 74–76, 82, 83, 86]), and only three included people with a transfemoral amputation (30%, n = 3 [74–76]). The mean sample size equalled 9. The remaining studies compared active with passive prostheses in people with a transtibial amputation (24%, n = 8) with a mean sample size of 9 [52, 55–58, 65, 77, 79].

**Table 2:** Study characteristics

Author	Participants	Prosthetic comparison	Familiarisation	Task	Outcome	Results
Arifin et al. 2014a	10 TTA • Age: 44.8 ± 13.5 yr • Gender: M = 10, F = 0 • Weight: 77 ± 17.9 kg • TSA: 7.1 ± 6.6 yr • MFCL: K2–K3 • AB-control group: **Y**/N • Cause of amputation: TR = 4, VA = 5, TU = 1	Passive vs passive • SACH (P) • Talux (P) • Single axis (P)	1 week	Standing Standing on BSS platform in 3 conditions (with rigid, compliant & unstable surface) for 20 s per condition	Biomechanical • Overall stability index • Anterior stability index • Posterior stability index • Medial stability index • Lateral stability index	• OSI, APSI, and MLSI indices were not affected by the interaction between prosthetic foot types and surface conditions • OSI ↑ using Talux ↔ SACH on foam surface ↔ firm and unstable support surface (p = 0.04) • Trend of stability indexes: lowest for SACH foot and highest for Talux foot in most of the conditions
Arifin et al. 2014b	10 TTA • Age: 44.8 ± 13.5 yr • Gender: M = 10, F = 0 • Weight: 77 ± 17.9 kg • TSA: 7.1 ± 6.6 yr • MFCL: K2–K3 • AB-control group: Y/**N** • Cause of amputation: TR = 4, VA = 5, TU = 1	Passive vs passive • SACH (P) • Talux (P) • Single axis (P)	2 weeks	Standing Standing on BSS platform in two conditions (eyes open & eyes closed) for 20 s per condition	Biomechanical • Overall stability index • Anterior stability index • Posterior stability index • Medial stability index Subjective • ABC-scale	• Control of postural steadiness unaffected by type of prostheses • MLSI > APSI for Talux in both eyes-opened and eyes-closed conditions (p = 0.034 and p = 0.017, respectively) • OSI, APSI and MLSI score > during eyes-closed ↔ eyes-opened condition for all foot types. Differences between the two conditions were only statistically significant in OSI (p = 0.018) and MLSI (p = 0.018) for SACH foot, as well as in OSI (p = 0.043) and APSI (p = 0.027) for Talux foot • ABC-scale: differences occurred between Talux and SACH (p = 0.043) as well as Talux and single axis foot (p = 0.028)
Childers et al. 2018	5 TTA • Age: 44 ± 13.9 yr • Gender: – • Weight: 80.5 ± 13.9 kg • TSA: 11.2 ± 5.3 yr • MFCL: – • AB-control group: Y/**N** • Cause of amputation: –	Passive vs passive • Proflex (P) • Variflex (P)	5 min	Treadmill slope walking 1 min of level, incline and decline walking at 1.1 m/s	Biomechanical • Foot angle • Prosthetic ankle and foot power • Whole body COM rate of energy change	• Range of motion ↑ with Pro-Flex foot • Energy return ↑ with Pro-Flex foot • Energy return from Pro-Flex foot ↓ ↔ sound limb ankle–foot system • Energy from Pro-Flex foot affected whole body COM mechanics • ↓ loading on sound limb = unclear
D'Andrea et al. 2014	8 TTA • Age: 47 ± 8 yr • Gender: M = 8, F = 0 • Weight: 98.6 ± 9.7 kg • TSA: 19.4 ± 11.8 yr • MFCL: ≥ K3 • AB-control group: **Y**/N • Cause of amputation: TR = 8	Active vs passive • Biom prototype (A) • Participants’ current prosthesis (P)	1 session with Biom of at least 2 h	Level walking Walking 3 times at 0.75, 1.00, 1.25, 1.50, and 1.75 m/s along 10-m walkway	Biomechanical • Whole-body angular momentum	During the affected leg stance phase • Sagittal whole-body angular momentum ranges > passive prostheses ↔ active prosthesis at 1.25 m/s (ES = 0.25; CI = 0.039–0.047, 0.037–0.045; p = 0.032) and 1.50 m/s (ES = 0.22; CI = 0.034–0.042, 0.031–0.039; p = 0.032) During the unaffected leg stance phase: • Sagittal whole-body angular momentum ranges > passive prosthesis at 0.75 m/s (ES = 0.33; CI = 0.046–0.060, 0.042–0.054; p = 0.031) and 1.75 m/s (ES = 0.33; CI = 0.023–0.031, 0.019–0.027; p = 0.017) ↔ active prosthesis • No differences in frontal whole-body angular momentum ranges between prostheses. no differences in transverse H at any speed, except for 0.75 m/s, transverse H range > passive prosthesis ↔ active prosthesis (ES = 0.11; CI = 0.016–0.026, 0.015–0.025; p = 0.040)
Darter et al. 2014	6 TTA • Age: 30 ± 4 yr • Gender: M = 5, F = 1 • Weight: 85.4 ± 16.9 kg • TSA: 2.8 ± 1.2 yr • MFCL: ≥ K2 • AB-control group: Y/**N** • Cause of amputation: –	Quasi-passive vs passive • Proprio (QP) • Participants’ current prosthesis (P)	3 weeks	Treadmill slope walking Walking at 3 speeds (0.89, 1.11, and 1.34 m/s) at each of three slope conditions (− 5°, 0°, and 5°)	Physiological • VO_2_ Subjective • RPE	• EE for walking with the current foot was 13.5% > for slope descent ↔ Proprio (on-mode) (p < 0.05) and 10.3% more than with the Proprio (off-mode) (p < 0.05) • No differences were found for EE during level walking and slope ascent • Mean energy cost values ↓ (improved economy) as speed ↑ during slope descent and level grade walking • Prosthetic foot type = significant (p < 0.01) during slope descent → less-economical gait with current prosthesis ↔ Proprio devices [Proprio (on-mode) 14.0%, p < 0.01, Proprio (off-mode) 10.5%, p < 0.05] but no differences between Proprio (on-mode) and Proprio (off-mode) • Perceived difficulty of walking ↑ as walking speed ↑ with significant device effect for slope descent (p < 0.01). RPE values ↓ with the Proprio (on-mode) by an average of 2.2 on the 6–20 scale ↔ current prosthesis (p < 0.01) and 1.8 with the Proprio (off-mode) ↔ current prosthesis (p < 0.01)
Davot et al. 2021	5 TTA • Age: 37.2 ± 15.2 yr • Gender: M = 4, F = 1 • Weight: 76.2 ± 12.2 kg • TSA: 3.4 ± 2.2 yr • MFCL: ≥ K2 • AB-control group: Y/**N** • Cause of amputation: –	Quasi-passive vs passive • Proprio (QP) • Meridium (QP) • Elan (QP) • Participants’ current prosthesis (P)	2 weeks	Level walking + slope walking 3 walking conditions at SS speed: on level ground, on a 12% (7°) ramp ascent and on a 12% (7°) ramp descent of 6.2 m long	Biomechanical • ROM • Equilibrium point • Hysteresis (= net energy loss of the system, computed on the entire gait cycle) • Late stance energy released • Quasi-stiffness	• ROM = Elan lowest maximal dorsiflexion in ascent (9°) and maximal plantarflexion in descent (12°). Dorsiflexion differences Meridium ↔ Elan (p = 0.008) and ↔ ESR (p = 0.0027). In every situation, the highest ROM was observed with the Meridium (mean = 19.5° in descent, 20.5° on level ground, 22.6° in ascent) and the lowest ROM with the Elan (mean = 18.9° in descent, 18.9° on level ground and 13.9° in ascent) • Equilibrium point of current prosthesis was similar in the three conditions (no shift of the curve along the X axis). For the Elan, the equilibrium point was not shifted for the first characteristic pattern. For the proprio, a shift could be observed between level ground and ascent; for the Meridium, between level ground and descent + between level ground and ascent • Hysteresis = Proprio and the current prosthesis presented lowest hysteresis in all conditions. The Meridium hysteresis was 2–3 times higher ↔ other 3 feet (p = 0.001) • Elan: the energy released was the lowest in descent and the highest in ascent. On level ground, it was ↑ ↔ descent and↓ ↔ ascent. Meridium had the lowest energy for propulsion • Quasi-stiffness = no differences between devices
De Asha et al. 2014	11 TTA 7 TFA • Age: 45 ± 12.4 yr • Gender: – • Weight: • TTA: 84.5 ± 17.0 kg • TFA: 86.3 ± 15.3 kg • TSA: 14.5 ± 14.4 yr • MFCL: ≥ K3 • AB-control group: Y/**N** • Cause of amputation: TR = 16, TU = 3	Passive vs passive • Echelon (P) • Participants’ current prosthesis (P)	No familiarisation	Level walking Walking 8 m–walkway at SS speed	Biomechanical • COM • COP • Swing time • Stance time • Inter-limb asymmetry • Step length Performance • Speed	• Walking speed = ↑ with Echelon and ↑ for TTA ↔ TFA • Aggregate negative CoP displacement was ↓ with Echelon. The CoP passed anterior to the shank earlier in stance with the Echelon • Instantaneous COM speed at intact-limb TO was unchanged across foot conditions but instantaneous COM speed minimum during the subsequent prosthetic-limb single support phase was ↑ using the Echelon. As a result, there was less slowing of COM speed (walking speed) during prosthetic-limb single support for both groups when using the Echelon ↔ current prosthesis. Peak COM speed during prosthetic limb stance was unchanged across foot conditions. All instantaneous COM speed values were ↑ for TTA ↔ TFA (p ≤ 0.045) • Swing time was longer for the prosthetic limb ↔ intact-limb and the differences between limbs was ↑ for TFA ↔ TTA • Stance time ↑ intact-limb ↔ prosthetic-limb & differences between limbs ↑ TFA ↔ TTA • Step length ↑ prosthetic limb ↔ intact limb • There were no effects of foot condition (p = 0.84) or group (p = 0.063) on cadence. There were no effects of foot condition on inter-limb asymmetry in swing time, stance time or step length. Swing and stance time inter-limb asymmetry were ↑ TFA ↔ TTA but there was no group effect on step length inter-limb symmetry
De Pauw et al. 2018	6 TTA 6 TFA • Age: • TTA: 54 ± 14 yr • TFA: 53 ± 14 yr • Gender: M = 11, F = 1 • Weight: • TTA: 80 ± 13 kg • TFA: 89 ± 16 kg • TSA: – • MFCL: K2–K4 • AB-control group: **Y**/N • Cause of amputation: –	Quasi-Passive vs passive • AMP-foot 4.0 (QP) • Participants’ current prosthesis (P)	No familiarisation	Treadmill walking 6-min treadmill walking at SS speed, 2-min slow and 2 min fast walking	Physiological • HR • MV • VO_2_ • VCO_2_ • RQ • METS Subjective • QUEST • RPE	• At normal speed, no significant differences between groups for MV, VO_2_, VCO_2_, RQ, and METS. In TTA, RQ ↑ with AMPfoot ↔ current prosthesis (p = 0.017). At other walking speeds, no differences were found • HR = At fast speed, no differences. At slow speed, HR ↑ in TFA and TTA with AMPFoot ↔ current prosthetic device. In TFA, HR ↑ with current prosthesis and AMP-foot ↔ able-bodied individuals (p = 0.043 and 0.008, respectively). At other speeds, no significant differences were revealed • At normal speed, RPE levels ↑ in TFA and TTA with current prosthesis and AMPFoot ↔ able-bodied individuals at the first (p ≤ 0.016 and p ≤ 0.004) and sixth minute (p ≤ 0.003 and p ≤ 0.004, respectively). No differences were observed between TFA and TTA when wearing the current prosthesis. RPE ↑ with AMPFoot in TFA ↔ TTA (p = 0.027). At slow and fast walking speeds, RPE ↑ for TFA and TTA ↔ able-bodied individuals for current prosthesis and AMPfoot (slow speed: p ≤ 0.004 and p ≤ 0.003, respectively; fast speed: p ≤ 0.005 and p ≤ 0.009, respectively). No differences in RPE were observed between TFA and TTA. In addition, at fast speed RPE ↓ in TTA ↔ TFA with AMPFoot (p = 0.042). In TFA, RPE levels were ↑ with AMPFoot ↔ current prosthesis at 1 and 6 min (p = 0.027 and 0.042, respectively) • QUEST = 10 participants responded positive regarding buying the device if it was available on the market. Only in TFA, significant lower values for satisfaction and weight of AMPFoot ↔ current prosthesis were observed (p = 0.038 and 0.042, respectively)
De Pauw et al. 2019	6 TTA 6 TFA • Age: • TTA: 54 ± 14 yr • TFA: 53 ± 14 yr • Gender: M = 11, F = 1 • Weight: • TTA: 80 ± 13 kg • TFA: 89 ± 16 kg • TSA: – • MFCL: K2–K4 • AB-control group: **Y**/N • Cause of amputation: –	Quasi-Passive vs passive • AMP-foot 4.0 (QP) • Participants’ current prosthesis (P)	No familiarisation	Treadmill walking Sustained Attention to Response Task, 6-min walking at SS speed + sustained attention to response task, 2-min walking at SS speed	Physiological • MRCP Performance • Dual-task accuracy	• Dual-task walking: reaction times ↑ for TFA with AMPfoot ↔ AB individuals (p = 0.020). During walking with AMPfoot significant accuracy differences of the no-go stimuli at the middle and end part of the cognitive task were observed • MRCP: no differences for MRCP amplitude and latency measures at electrode Cz between AB individuals and TTA walking with the current or novel prosthetic device. TFA walking with AMPfoot did not exhibit MRCPs, but TFA walking with the current prosthesis showed MRCPs at different electrode locations. No differences in activity of the brain sources of the different MRCP peaks were observed when TTA walked with the current and novel prosthetic device. Additionally, no significant differences were observed when TTA walked with the current prosthetic device ↔ AB individuals. On the other hand, ↔ AB individuals TTA wearing the AMPfoot showed ↑ activity of brain sources at the first positive deflection
De Pauw et al. 2020	6 TTA 6 TFA • Age: • TTA: 54 ± 14 yr • TFA: 53 ± 14 yr • Gender: M = 11, F = 1 • Weight: • TTA: 80 ± 13 kg • TFA: 89 ± 16 kg • TSA: – • MFCL: K2–K4 • AB-control group: **Y**/N • Cause of amputation: –	Quasi-Passive vs passive • AMP-foot 4.0 (QP) • Participants’ current prosthesis (P)	No familiarisation	Treadmill walking 2-min walking at SS speed, 2 min at slow (− 25% self-selected) and 2 min at fast (+ 25% self-selected) speeds. 1 min rest in between tasks	Biomechanical • LE joint angles • LE angular velocities • Stride length • Step width • Maximum GRF Performance • Speed	• TFA did not benefit from walking with the novel prosthesis • TTA walking at slow and normal speed with AMPfoot 4.0 → beneficial effects at the level of the ankle and knee • No differences between walking with the current prostheses and AMPfoot 4.0 with respect to force platform data
Delussu et al. 2016	20 TTA • Age: 66.6 ± 6.7 yr • Gender: M = 17, F = 3 • Weight: 78.5 ± 13.2 kg • TSA: – • MFCL: K1–K2 • AB-control group: Y/**N** • Cause of amputation: TR = 6, VA = 13, TU = 1	Passive vs passive • 1M10 (P) • SACH (P)	30 days	Level walking 6MWT along 30-m-long linear course	Physiological • MV • VO_2_ • RER • HR • REI • Energy cost Performance • SS speed Subjective • RPE • Satisfaction	• No differences for MV, VO_2_, RER, HR and REI using SACH or 1M10 • Energy cost, SS speed, RPE score and SATPRO improved with the 1M10 compared to the SACH
Esposito et al. 2014a	10 TTA • Age: 30.2 ± 5.3 yr • Gender: M = 9, F = 1 • Weight: 95.8 ± 7.3 kg • TSA: – • MFCL: ≥ K3 • AB-control group: **Y**/N • Cause of amputation: TR = 10	Active vs passive • BiOM (A) • Participants’ current prosthesis (P)	3 weeks	Level walking Walked at 3 controlled speeds	Biomechanical • GRF • Knee joint moments • Loading rate Subjective • Rating of ambulation ability Performance • Speed	• The active prosthesis did not ↓ sound limb’s peak adduction moment or its impulse, but did ↓ the external flexor moment, peak vertical force and loading rate as speed ↑ • The active prosthesis ↓ loading rate from AB controls. The sound limb did not display a greater risk for knee osteoarthritis ↔ intact limb or ↔ AB controls in either device • Self-selected walking speeds were not significantly different between prosthesis conditions • Subject rating of ambulation ability using the PEQ was high in both devices
Esposito et al. 2014b	6 TTA • Age: 23 ± 5 yr • Gender: M = 5, F = 1 • Weight: 91.4 ± 12.1 kg • TSA: – • MFCL: – • AB-control group: **Y**/N • Cause of amputation: TR = 6	Active vs passive • BiOM (A) • Participants’ current prosthesis (P)	3 weeks	Level walking + slope walking Walking at standardized speed (± 5) over level ground and inclined walkway + 6MWT on treadmill until steady state metabolic rate was achieved for both level and inclined walking	Biomechanical • Step-to-step transition work • LE joint angles • LE joint moments • LE joint power Physiological • Metabolic rate	• Kinetics & kinematics: during level walking, the BiOM ↑ peak ankle plantarflexion angles and powers at push-off ↔ current prosthesis and ↓ peak internal plantar flexor moments. During inclined walking, peak angles and powers ↑ in the BiOM. Peak ankle plantarflexion angles and powers ↓ in current prosthesis ↔ AB controls over level ground, and angles, moments, and powers ↓ on the incline. The BiOM normalized the peak ankle plantarflexion angles on level ground, but moments remained ↓ and powers ↑ ↔ AB controls during level ground and inclined walking • Metabolic rate: during level walking, VO_2_ was ↓ 16% with BiOM ↔ current prosthesis. ↑ 9% metabolic rates with current prosthesis ↔ able-bodied individuals, but BiOM normalized metabolic rates. On the incline, metabolic rates were not different between BiOM ↔ AB controls or between BiOM ↔ current prosthesis • Step-to-step transition = During level walking, the net step-to-step transition work prosthetic limb ↑ 63% with active ↔ current prosthesis. Active prosthetic trailing limb step-to-step transition work ↑28% ↔ AB controls, while current prosthesis ↓ 22% ↔ AB controls • Net leading limb work during step-to-step transitions inclined walking ↑ 53% with active prosthesis ↔ current. Net trailing limb step-to-step transition work did not differ between AB controls and TTA
Ferris et al. 2012	11 TTA • Age: 29.8 ± 5.3 yr • Gender: M = 10, F = 1 • Weight: 95 ± 7.3 kg • TSA: – • MFCL: – • AB-control group: **Y**/N • Cause of amputation: TR = 11	Active vs passive • BiOM (A) • Participants’ current prosthesis (P)	3 weeks	Level walking + agility and mobility Walking at SS and controlled speed + T-test, Four square step test, Hill and stair Assessment Tests	Biomechanical • GRF • Symmetry • Stance time • Swing time • Cadence • Step length • Stride length • LE joint angles • LE joint moments • LE joint powers Performance • Agility and mobility • Speed Subjective • User satisfaction	• Ankle ROM 30% > active prosthesis ↔ AB current, both < ROM ↔ AB control and intact limbs • Peak ankle power ↓ 40% current prosthesis ↔ active • Peak knee power ↑ 35% active prosthesis ↔ AB control ↑ 125% current → active absorbing 2 × the peak knee power observed in AB control and intact limbs • Peak hip power ↑ 45% active prosthesis ↔ intact limb • Walking speed ↑ active prosthesis ↔ current (not significant) ↔ AB control group • User satisfaction scores → preference for active over current prosthesis
Gailey et al. 2012	10 TTA • Age: • Group 1: 60.6 ± 2.3 yr • Group 2: 51 ± 5.8 yr • Gender: M = 9, F = 1 • Weight: • Group 1: 105.5 ± 6.4 kg • Group 2: 92.1 ± 9.7 kg • TSA: • Group 1: 2.90 ± 1.8 yr • Group 2: 16.1 ± 17.6 yr • MFCL: K2–K3 • AB-control group: Y/**N** • Cause of amputation: TR = 5, VA = 5	Quasi-Passive vs passive • SACH (P) • SAFE foot (P) • Talux (P) • Proprio (QP) • Participants’ current prosthesis (P)	2 weeks	Level walking Performing LCI-5, 6MWT	Performance • LCI-5 • 6MWT • Steps/day • AMPRO • Hours of daily activity Subjective • PEQ-13	• PEQ-13, LCI-5, 6MWT, or step activity monitor: no differences between devices • AMPPRO: differences following training with the existing prosthesis in group 1 and between selected feet from baseline testing (p ≤ 0.05). Sign differences were found between group 1 and group 2 (p ≤ 0.05) in the AMPPRO and 6MWT when using the Proprio foot • Self-report measures were unable to detect differences between prosthetic feet
Gardinier et al. 2017	10 TTA • Age: 46.6 ± 15 yr • Gender: M = 10, F = 0 • Weight: 93.2 ± 17.9 kg • TSA: – • MFCL: K3–K4 • AB-control group: **Y**/N • Cause of amputation: TR = 9, VA = 1	Active vs passive • BiOM (A) • Participants’ current prosthesis (P)	8 min	Treadmill walking Walking along 8-m walkway at at SS speed and controlled speed + walking 8-min on treadmill until steady-state energy expenditure is reached	Physiological • Energetic cost • VO_2_ • Cost of transport Performance • Speed	• No sign differences in VO_2_ (2.9% difference; P = 0.606, d = 0.26) using the active ankle ↔ current prosthesis • No sign differences in cost of transport (1% difference; P = 0.652, d = 0.23) using the active ankle ↔ current prosthesis • No sign differences in preferred walking speed (1% difference; P = 0.147, d = 0.76) using the active ankle ↔ current prosthesis • Participants classified as having the highest function (MFCL = K4) were sign more likely to exhibit energy cost savings ↔ those classified as having lower function (K3; P = 0.014, d = 2.36)
Gates et al. 2013	11 TTA • Age: 30 ± 5 yr • Gender: M = 10, F = 1 • Weight: 95 ± 7.3 kg • TSA: – • MFCL: – • AB-control group: Y/**N** • Cause of amputation: TR = 11	Active vs passive • BiOM (A) • Participants’ current prosthesis (P)	3 weeks	Walking (rocky surface) Walking across a loose rock surface at three controlled speeds; The rock surface was a 4.2-m long by 1.2-m wide by 10-cm deep pit filled with loose river rocks from a major hardware store	Biomechanical • COM • Minimum margin of stability Performance • Speed	• Walking speed ↑ 10% using active prostheses (1.16 m/s) ↔ current (1.05 m/s; p = 0.031) • Ankle plantarflexion ↑ (p < 0.001), knee flexion ↓ (p = 0.045) on their prosthetic limb using active prostheses ↔ current • Other kinematics of the knee and hip = nearly identical between devices • Medial–lateral motion COM ↓ using active prosthesis ↔ current (p = 0.020), • Medial–lateral margins of stability = no differences between devices (p = 0.662)
Grabowski et al. 2013	7 TTA • Age: 45 ± 6 yr • Gender: – • Weight: 99.5 ± 10.2 kg • TSA: 21.1 ± 11.3 yr • MFCL: ≥ K3 • AB-control group: Y/**N** • Cause of amputation: –	Active vs passive • Active prototype (A) • Participants’ current prosthesis (P)	2 h	Level walking Walking at 0.75, 1.00, 1.25, 1.50, and 1.75 m/s along 10 m-walkway	Biomechanical • GRF • Knee joint moments • Loading rates	• Active prosthesis ↓ unaffected leg peak resultant forces by 2–11% at 0.75–1.50 m/s ↔ current • Active prosthesis ↓ first peak knee external adduction moments by 21 and 12% at 1.50 and 1.75 m/s ↔ current • Loading rates = no differences between prostheses
Graham et al. 2007	6 TFA • Age: 40.3 ± 6.3 yr • Gender: – • Weight: 88.5 ± 9.4 kg • TSA: – • MFCL: – • AB-control group: Y/**N** • Cause of amputation: –	Passive vs passive • VariFlex (P) • Multiflex (P)	3–6 weeks	Level walking Timed walking test along 207.3-m oval circuit including outdoor and indoor walking with the resultant variations of camber and surface	Biomechanical • Step-length ratio • Stance time • Vertical GRF • Ankle dorsiflexion • Knee flexion • Hip flexion/extension • Transverse pelvic rotation • Ankle power • Hip power Performance • Speed Subjective • Prosthetic socket fit comfort score	• VariFlex speed ↑ + ↑ equal step lengths at fast speed ↔ multiflex • VariFlex ↑ peak ankle dorsiflexion at push-off on the prosthetic side (18.3° + − 4.73°, P < 0.001) + ↑ 3 × power from the prosthetic ankle at push-off (1.13 + − 0.22 W/kg, P < 0.001) ↔ multiflex • No sign differences in temporal symmetry or loading of the prosthetic limb, in the timed walking test with each foot, or in the comfort score
Graham et al. 2008	6 TFA • Age: 40.3 ± 6.3 yr • Gender: – • Weight: 88.5 ± 9.4 kg • TSA: – • MFCL: – • AB-control group: Y/**N** • Cause of amputation: –	Passive vs passive • VariFlex (P) • Mutliflex (P)	3–10 weeks	Treadmill walking 2-min walking tests; Speeds increases every 2 min starting at 0.83 m/s then 0.94 m/s, 1.1 m/s, 1.25 m/s, 1.39 m/s, 1.53 m/s, 1.67 m/s and 1.81 m/s until subjects find the treadmill speed too fast	Physiological • Mean VO_2_ Performance • Speed	• VariFlex ↓ mean VO_2_ ↔ Multiflex at all speeds, although the differences were only sign at speeds of 0.83 and 1.1 m/s. The estimated differences across all speeds was 3.54 mL/kg min
Heitzmann et al. 2018	11 TTA • Age: 37.9 ± 12.3 yr • Gender: M = 9, F = 2 • Weight: 81.1 ± 17.4 kg • TSA: 11.9 ± 10.6 yr • MFCL: K3–K4 • AB-control group: **Y**/N • Cause of amputation: TR = 4, VA = 2, TU = 4, O = 1	Passive vs passive • Proflex pivot (P) • Participants’ current prosthesis (P)	30–45 min	Level walking Walking along 10 m-walkway at SS speed	Biomechanical • Ankle ROM • Peak ankle moment, peak ankle power • Peak external knee varus moment • Peak vertical GRF Performance • Speed	• Proflex ↓ walking speed (1.33 ± 0.16 m/s) ↔ current prosthesis (1.39 ± 0.17 m/s). AB controls did not walk sign faster ↔ TTA • Proflex ↑ prosthetic ankle ROM by 12.5° ↔ current prosthesis • Angle ROM and peak dorsiflexion of 18.8° ↔ current prosthesis + no sign differences ↔ AB controls • Peak external ankle dorsi-flexion moment < AB controls (proflex: 28%, current prosthesis: 36% + no sign differences in peak external ankle dorsi-flexion moment between prosthetic feet • Peak positive ankle power < current prosthesis (by 66%) and Proflex (by 33%) ↔ AB controls + Proflex ↑ peak ankle power ↔ current prosthesis • External knee varus moment and the peak vertical GRF for Proflex ↓ ↔ current prosthesis & AB controls
Houdijk et al. 2018	15 TTA • Age: 58.8 ± 11.1 yr • Gender: – • Weight: 86 ± 12.6 kg • TSA: – • MFCL: K3 • AB-control group: Y/**N** • Cause of amputation: TR = 12	Passive vs passive • SACH (P) • Variflex (P)	1 day	Level walking Walking along 10-m walkway at a fixed speed	Biomechanical • Work • Vertical COM • Step length intact • Step length symm • Backward Margin of stability	• Push-off work ↑ Variflex ↔ SACH • COM speed at toe-off ↑ Variflex ↔ SACH • Intact step length and step length symmetry ↑ without ↓ the backward margin of stability Variflex ↔ SACH
Hsu et al. 2006	8 TTA • Age: 36 ± 15 yr • Gender: M = 8, F = 0 • Weight: 81.7 ± 9.6 kg • TSA: – • MFCL: – • AB-control group: Y/**N** • Cause of amputation: –	Passive vs passive • C-walk (P) • Flex foot (P) • SACH (P)	4 weeks	Treadmill walking 2 min walking at SS speed	Physiological • Gait efficiency • VO_2_ • %APMHR Performance • Steps/day Subjective • RPE	• C-Walk had a trend of ↑ physiologic responses ↔ SACH • Flex foot: no sign differences in EE and gait efficiency, but ↓ %APMHR & RPE ↔ C-Walk and SACH
Johnson et al. 2014	21 TTA • Age: 48.2 ± 12.8 yr • Gender: M = 18, F = 3 • Weight: 87.4 ± 13.2 kg • TSA: 8.8 ± 14 yr • MFCL: – • AB-control group: Y/**N** • Cause of amputation: –	Passive vs passive • Echelon (P) • Participants’ current prosthesis (P)	45 min	Level walking Walking along 8 m-walkway	Biomechanical • MTC • LE joint angles • Prosthetic limb hip-hiking Performance • Speed	• Mean MTC ↑ on both limbs with Echelon ↔ current prosthesis (p = 0.03) • Walking speed ↑ Echelon ↔ current prosthesis (p = 0.001) + ≈ ↑ swing-limb hip flexion on the prosthetic side Echelon ↔ current prosthesis (p = 0.04) • Variability in MTC ↑ on the prosthetic side with Echelon (p = 0.03), but this did not ↑ risk of tripping
Prakash et al. 2020	15 TTA • Age: 33.3 ± 5.5 yr • Gender: – • Weight: – • TSA: – • MFCL: – • AB-control group: Y/**N** • Cause of amputation: –	Passive vs passive • SACH (P) • Passive prototype ESR (P)	15 min	Level walking 10‑m walk test + 5 min of strolling at SS speed	Biomechanical • Stride length • Cadence Physiological • PCI Performance • Speed	• Stride length, cadence, speed, and PCI ↓ SACH ↔ current prosthesis
Paradisi et al. 2015	20 TTA • Age: 66.7 ± 6.7 yr • Gender: M = 17, F = 3 • Weight: 78.7 ± 13.2 kg • TSA: 9.8 ± 13.5 yr • MFCL: – • AB-control group: Y/**N** • Cause of amputation: TR = 6, VA = 13, O = 1	Passive vs passive • 1M10 (P) • SACH (P)	4 weeks	Level walking, slope walking and stair climbing Performing 6MWT, LCI-5, HAI, SAI, BBS	Performance • Score on BBS, LCI-5, HAI, SAI • Time • Speed • Upright Gait Stability Subjective • PEQ	• Walking speed ↑ 1M10 ↔ SACH (p < 0.05) maintaining the same upright gait stability • BBS, LCI-5, and SAI times and 4 of 9 subscales of the PEQ ↑ 1M10 ↔ SACH
Rábago et al. 2016	10 TTA • Age: 30.2 ± 5.3 yr • Gender: M = 9, F = 1 • Weight: 96.1 ± 6.8 kg • TSA: – • MFCL: – • AB-control group: **Y**/N • Cause of amputation: –	Active vs passive • BiOM (A) • Participants’ current prosthesis (P)	3 weeks	Slope walking walking along 5 m long, 5˚ sloped ramp at controlled speed	Biomechanical • GRF • Stance time • Step length • Stride length • Swing time • LE joint angles • LE joint moments and powers • Second vertical peak • Braking • Propulsion Performance • Speed	• During slope ascent, the BiOM ↑ prosthetic ankle plantarflexion and push-off power generation ↔ current prosthesis + matched AB controls more closely • Similar deviations and compensations between both feet • Transitioning off the prosthetic limb → ↑ ankle plantarflexion and push-off power with BiOM → ↓ intact limb knee extensor power production → ↓ demand on the intact limb knee ↔ current prosthesis
Riveras et al. 2020	13 TTA • Age: 38.2 ± 13.2 yr • Gender: M = 10, F = 3 • Weight: 75.1 ± 15.4 kg • TSA: 10.8 ± 13.1 yr • MFCL: – • AB-control group: Y/**N** • Cause of amputation: TR = 10, VA = 2, O = 1	Passive vs quasi-passive • Esprit (P) • Echelon (QP) • Elan (QP)	1 h	Slope walking walking along 6 m, 5° inclination ramp at SS speed	Biomechanical • Tripping probability • Coefficient of variation • Minimum toe clearance	• MTC median values for ascending (P ≤ 0.001, W = 0.58) and descending the ramp (P = 0.003, W = 0.47) in the prosthetic limb ↑ Elan ↔ Esprit and Echelon • CV ↓ on the prosthetic limb for descending the ramp (P = 0.014, W = 0.45) using the Echelon and Elan ↔ Esprit • Elan = Lowest TP for the prosthetic leg in three conditions evaluated • On the sound limb results showed the median MTC was ↑ (P = 0.009, W = 0.43) and CV ↓ (P = 0.005, W = 0.41) during ascent using Echelon and Elan ↔ Esprit
Schmalz et al. 2019	4 TTA • Age: 56 ± 12 yr • Gender: M = 4, F = 0 • Weight: 79 ± 8.0 kg • TSA: – • MFCL: K3 – K4 • AB-control group: Y/**N** • Cause of amputation: TR = 3, VA = 1	Passive vs quasi-passive • Meridium (QP) • Participants’ current prosthesis (P)	2 weeks	Slope walking Walking along circuit of 3 m downhill walkway (10° inclination) followed by specific uphill and downhill elements with opposite inclination angles of 10	Biomechanical • GRF • LE joint moments • LE joint angles	• Meridium ↑ ankle adaptation to the abruptly changing inclination, reflected by a ↑ stance phase dorsiflexion ≈ to AB controls ↔ current prosthesis • Peak value of the knee extension moment on the prosthetic side was ↑ with current prosthesis, whereas it was almost normal with Meridium (current prosthesis: 0.71 ± 0.13 Nm/kg, Meridium: 0.42 ± 0.12 Nm/kg, NA: 0.36 ± 0.07 Nm/kg, p < 0.05 and p < 0.01) • External knee adduction moment was ↓ for TTA and did not show differences between prostheses
Segal et al. 2015	7 TTA • Age: 52.3 ± 12 yr • Gender: – • Weight: 80.9 ± 9.9 kg • TSA: – • MFCL: – • AB-control group: **Y**/N • Cause of amputation: TR = 7	Passive vs quasi-passive • Participants’ current prosthesis (P) • Lightfoot2 (P) • Prototype: Controlled Energy Storage and Return prosthetic foot (QP)	5 min	Level walking Walking on a treadmill at the target speed of 1.14 m/s for 10 min, until they reached steady state. + walking along a 10 m-walkway at same speed	Biomechanical • GRF • COM • LE joint powers • Work during gait Physiological • VO_2_	• ↑ energy storage during early stance, ↑ prosthetic foot peak push-off power and work, ↑ prosthetic limb COM push-off work and ↓ intact limb COM collision work with Controlled Energy Storage and Return prosthetic foot ↔ Lightfoot2 and current prosthesis • Biological contribution of the positive COM work for Controlled Energy Storage and Return prosthetic foot was ↓ ↔ Lightfoot2 and current prosthesis • Net metabolic cost for Controlled Energy Storage and Return prosthetic foot did not change comp ↔ Lightfoot2 and ↑ ↔ current prosthesis
Struckov et al. 2016	9 TTA • Age: 41.2 ± 12.9 yr • Gender: M = 9, F = 0 • Weight: 74.1 ± 15.7 kg • TSA: – • MFCL: K3 • AB-control group: Y/**N** • Cause of amputation: –	Passive vs quasi-passive • Elan (QP) • Epirus (P)	20 min	Slope walking Ramp descent at slow and customary speed	Biomechanical • Residual-knee loading, response flexion • Single-support minimum flexion • Time to foot flat • CoP • Prosthetic-limb shank mean angular velocity during single-support • Single-support residual-knee moment impulse • Single-support negative mechanical work at the residual hip and knee joints • Unified deformable segment	• Foot-flat was attained fastest with the Epirus and second fastest with the Elan (P < 0.001) • Prosthetic shank single-support mean rotation speed ↓ (p = 0.006), flexion (P < 0.001) ↓, negative work done at the residual knee (P = 0.08) ↓, and negative work done by the ankle–foot ↑ (P < 0.001) with Elan ↔ Epirus and Elan in off-mode
Underwood et al. 2012	11 TTA • Age: 42.5 ± 13.5 • Gender: M = 8, F = 3 • Weight: 80.3 ± 14.3 kg • TSA: 11.1 ± 13.3 yr • MFCL: – • AB-control group: Y/**N** • Cause of amputation: –	Passive vs passive • FlexWalk (P) • SAFE FOOT 2 (P)	30 min	Level walking Walking along 10 m-walkway at SS speed	Biomechanical • LE peak joint moments and power • Perceived stability and mobility	• The majority of the kinetic differences that occurred due to the changing of prosthetic foot type were limited to ankle joint variables in the sagittal plane with ↑ peak moments and power during propulsion for the Flex foot ↔ SAFE foot • Effects were also found at joints proximal to the prosthesis (e.g., knee) and differences were also found in the kinetics of the sound limb
Wezenberg et al. 2014	15 TTA • Age: 55.8 ± 11.1 yr • Gender: M = 15, F = 0 • Weight: 86 ± 12.6 kg • TSA: – • MFCL: – • AB-control group: Y/**N** • Cause of amputation: TR = 15	Passive vs passive • SACH (1D10) (P) • Variflex (P)	1 day	Level walking Walking along 10 m-walkway at SS speed	Biomechanical • GRF • COM mechanical work • Work at push-off • COP • Step length • Symmetry Performance • Speed	• Positive mechanical work COM performed by the trailing prosthetic limb was ↑ (33%, p = 0.01) and the negative work performed by the leading intact limb ↓ (13%, p = 0.04) with Variflex ↔ SACH foot • ↓ step-to-step transition cost & ↑ mechanical push-off power and extended forward progression of the COP with Variflex ↔ SACH
Yang et al. 2017	10 TTA • Age: 63.8 ± 2.5 yr • Gender: M = 10, F = 0 • Weight: – • TSA: 3.1 ± 0.8 yr • MFCL: K2–K3 • AB-control group: Y/**N** • Cause of amputation: –	Passive vs passive • 1C30 Trias (P) • 1C60 Triton (P)	1 week	Level walking Walking along 10 m-walkway at SS speed	Biomechanical • Cadence • Step width • Step length • Stance and swing phase ratio • LE joint angles • Ankle plantarflexion moment at end of stance Performance • Speed	• Cadence asymmetry with Trias was observed. Ankle plantarflexion at the end of stance and ankle supination at the onset of pre-swing ↓ with both prosthetic feet ↔ intact side. Other spatiotemporal, kinematic, and kinetic data showed no sign differences in a side-to-side comparison • In a comparison between the two prosthetics, stance and swing ratio and ankle dorsiflexion through mid-stance was closer to normal with Triton ↔ Trias. Other spatiotemporal, kinematic, and kinetic data showed no statistically sign differences between prosthetics

%APMHR = percentage of age-predicted maximum heart rate, 6MWT = 6-min’ walk test, A = active prosthesis, AB = able-bodied, ABC-scale = activities-specific balance confidence, AMPRO = Amputee Mobility Predictor Assessment Tool, APSI = anterior stability index, BBS = Berg Balance Scale, CI = confidence interval, COM = centre of mass, COP = centre of pressure, CV = coefficient of variation, EE = energy expenditure, ES = effect size, GRF = ground reaction force, H = whole-body angular momentum, HAI = Hill Assessment Index, HR = heart rate, LCI-5 = Locomotor Capabilities Index, LE = lower extremity, METS = metabolic equivalents, MFCL = medicare functional classification, MLSI = medial stability index, MRCP= movement-related cortical potential, MTC = minimum toe clearance, MV = minute ventilation, O = other, OSI = overall stability index, P = passive prosthesis, PCI = Physiological Cost Index, PEQ = Prosthetic Evaluation Questionnaire, QP = quasi-passive prosthesis, QUEST = Quebec user evaluation of satisfaction with assistive technology, REI = Relative exercise intensity, RER = respiratory exchange ratio, RPE = rating of perceived exertion, RQ respiratory quotient, SACH = solid ankle cushioned heel, SAI = Stair Assessment Index, Sign Significant, SS = self-selected speed, symm symmetry, TFA = individual with unilateral transfemoral amputation, TR = trauma, TSA = time since amputation, TTA = individual with unilateral transtibial amputation, TU = tumour, VA = vascular, VCO_*2*_ = carbon dioxide production, VO_*2*_ = oxygen consumption, yr = year

The prosthetic evaluation was mainly conducted through level walking tasks. Within the cluster of studies comparing passive prostheses, level walking tasks amounted to 63% (n = 10) [54, 61–64, 68, 73, 81, 84, 85]. Among those comparing passive to quasi-passive ankle–foot devices, the number equalled 30% (n = 3) [72, 82, 86]. For those comparing active to passive devices, level walking was assessed in 63% (n = 5) [52, 55–57, 79]. Less frequent tasks included treadmill level walking (19%, n = 3 [60, 71, 80]; 30%, n = 3 [74–76] and 13%, n = 1 [77], respectively), slope walking (6%, n = 1 [63]; 40%, n = 4 [66, 67, 72, 83] and 25%, n = 2 [55, 65], respectively) and treadmill slope walking (6%, n = 1 [71]; 10%, n = 1 [53] and 0%, n = 0, respectively). Tasks that were only performed to a limited extent were, within studies comparing passive prostheses, standing tasks (13%, n = 2) [69, 70], completing a walking circuit (6%, n = 1) [59] and climbing stairs (6%, n = 1) [63]. Within studies comparing active with passive prostheses, these were walking over rocks and performing clinical tests assessing agility and mobility (13%, n = 1 [58] and 13%, n = 1 [57], respectively).

### Therapeutic benefits

All studies investigated the short-term effects of performing daily activities with prosthetic ankle–foot devices, and none of the studies examined long-term effects. The mean familiarisation time amounted to 11 days (range: no familiarisation–6 weeks). Biomechanical outcome measures were most frequently gathered. Within the cluster of studies comparing passive prostheses, the number of biomechanical measures amounted to 81% (n = 13) [59, 61, 62, 64, 68–71, 73, 80, 81, 84]. Among these, 11 found results favouring the newly tested passive prosthesis [59, 61, 62, 64, 68, 69, 71, 73, 81, 84]. Within the studies comparing passive with quasi-passive prostheses, the number of biomechanical measures was 60% (n = 6), and all reported positive results favouring the quasi-passive device [66, 67, 72, 76, 82, 83]. Within the studies comparing active with passive prostheses, 88% (n = 7) [52, 55–58, 65, 79] reported biomechanical outcome measures, all found positive effects in favour of the active device, and 1 article also found a negative effect [55].

Within studies comparing passive prostheses, comparing passive with quasi-passive prostheses, and comparing active with passive prostheses, physiological outcome measures (25%, n = 4 [54, 60, 64, 80]; 40%, n = 4 [53, 75, 76, 82] and 25%, n = 2 [55, 77] respectively) and performance outcomes were less frequently investigated (69%, n = 11 [54, 59–61, 63, 64, 68, 73, 80, 81, 85]; 33%, n = 3 [75, 76, 86] and 63%, n = 5 respectively [56–58, 65, 77]). Within the cluster of studies comparing passive prostheses, four studies reported physiological benefits [50, 52, 55, 58], and eight studies reported benefits on the performance of the novel passive prosthesis [54, 59, 61, 63, 64, 73, 80, 81]. Among the studies comparing passive with quasi-passive prostheses, one study reported positive physiological effects for the quasi-passive prosthesis [53], one reported negative physiological effects [74], and one found favourable results on performance regarding the quasi-passive prosthesis [75]. Within the studies comparing active with passive prostheses, 1 study reported a physiological benefit [55], and two reported a benefit on performance with the active prosthesis [57, 58]. Lastly, subjective outcome measures were scarce (31%, n = 5 [54, 59, 63, 70, 84]; 30%, n = 3 [53, 74, 86] and 25%, n = 2 [56, 57] respectively). Three studies favoured the novel passive prosthesis within the cluster of studies comparing passive prostheses [54, 63, 70]. Among the studies comparing passive with quasi-passive prostheses, one found positive results [53], and mixed results for the quasi-passive device [74]. One study favoured the active device within the studies comparing active with passive prostheses [57].

## Discussion

The purpose of this study was to systematically review the therapeutic benefits of performing daily activities with passive, quasi-passive and active ankle–foot prostheses in people with a unilateral lower limb amputation. Remarkably, no studies investigated the long-term therapeutic benefits.

**Fig. 4:**
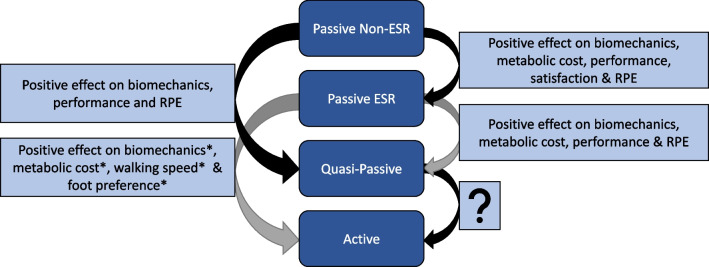
Short-term therapeutic benefits of passive, quasi-passive and active ankle–foot prostheses in people with a unilateral transtibial and transfemoral amputation. The arrows indicate the effect of switching from one type of prosthesis to another. For example, switching from passive non-ESR to quasi-passive prostheses entails positive effects on biomechanics, performance and RPE. ESR: energy-storing and release; RPE: rating of perceived exertion; ?: currently unknown, to be investigated; *effect based on studies only including people with a transtibial amputation

Figure 4 captures the short-term therapeutic benefits of passive, quasi-passive and active prostheses. This figure shows the domains in which benefits were found. It was not possible to provide such an overview at the outcome measure level due to high heterogeneity. Overall, the numerous outcome measures per study yielded positive results on biomechanical, physiological, performance-related or subjective outcomes for the more advanced prostheses, implying therapeutic benefits for the individuals walking with them, though all studies also identified no or unfavourable effects. The technological innovations contribute to improving the quality of life in the short-term when people with lower limb amputation switch the conventional passive cushion foot for a more advanced prosthesis (i.e. the passive energy-storing release feet, the surface-adaptive quasi-passive feet, the active feet generating an external force through an actuator). However, comparisons between active prostheses and quasi-passive devices have not yet been conducted.

Among the included studies, quality of life has been evaluated using biomechanical, physiological, performance-related or subjective measures. The biomechanical and physiological dimensions of quality of life have been assessed during level and slope walking in 94% of the included studies, while only 29% included the subjective dimension. In general, gait efficiency and efficacy improved in parallel with technological advances, though gait asymmetries remained [52–62, 64–73, 75–77, 79–84]. Further in-depth discussion of these parameters is not feasible due to the heterogeneity in outcome measure among the biomechanical and physiological parameters (Table 2). Nevertheless, it is the ultimate goal of prosthetic development to strive towards the most efficient gait patterns by seeking complete gait symmetry and matching the gait patterns as closely as possible to those of able-bodied individuals [37]. Furthermore, the limited data on the subjective dimension of quality of life revealed that the perceived effort and satisfaction increased in line with the advancement of the devices. The limited use of subjective measures can be attributed to the prohibitive cost of most active and quasi-passive devices for a subset of individuals. This factor might introduce a confounding variable in the data affecting subjective feedback. Conversely, these paywalls will not affect the biomechanical or physiological data. Nevertheless, subjective measures (e.g. perceived effort, satisfaction, feedback on the noise of motors in active prostheses) should be more prominent in prosthetic evaluations, as they are crucial to assessing the quality of life [87].

Due to its biomechanical focus, the prosthetic evaluation primarily targets aberrated movement patterns that can be remedied in the short term by a prosthesis [4, 6, 7]. However, movement patterns are orchestrated by the intertwining between biomechanical factors and the human brain [27, 88]. This entails that the brain plays a vital role in the organization and performance of human gait [88]. Magnetic resonance imaging revealed that amputation causes thinning of the premotor cortex and visual-motor area combined with a decrease in white matter integrity in the premotor area contralateral to the amputation and at a bilateral connection between both premotor cortices [27]. These changes interfere with movement planning or coordinating eye movements in relation to limbs and lead to decreased perception–action coupling [27]. Additionally, amputation causes changes in limb representation in the primary motor cortex and somatosensory cortex, and causes decreased connectivity between many brain areas, including the primary motor cortex, primary somatosensory cortex, basal ganglia, thalamus and cerebellum [27]. These changes in connectivity translate towards reduced motor control and balance and potentially lead to falls [9, 10, 27]. Remarkably, only a single study examining the effect on brain functioning across prosthetic ankle–foot prostheses has been included in this review [75]. De Pauw et al. [75] explored whether motor-related cortical potentials differed between passive and quasi-passive prostheses during daily activities using electro-encephalography but did not detect any difference between both devices. The absence of an effect is not unexpected, considering neuroplasticity is a time-consuming process, and sufficient familiarisation time was not provided [89–94]. Unravelling neuroplasticity in relation to the type of prosthesis may provide a new understanding of the effects of prostheses to improve the quality of life in people with a lower limb amputation.

A conceivable approach to account for the brain’s influence is through dual tasks, conditional on adequate familiarisation [95]. Dual tasks involve the concurrent performance of two tasks and are regarded as a measurement of cognitive-motor capacity as they require executive function and attentional demand [95]. Their performance usually results in decreased mobility and deteriorated gait patterns leading to increment falls [96, 97]. Out of the included articles in this review, only 1 investigated the difference between passive and quasi-passive prostheses during the performance of a dual-task during treadmill walking [75]. They found that only in individuals with a transfemoral amputation attention demands (reaction times and accuracy) increased during walking with the quasi-passive prosthesis compared to the current prosthesis and able-bodied individuals [75]. Lack of familiarization time to habituate to the new prosthetic device may have influenced these results. As discussed earlier, the negative implications of performing dual-tasks are attributable to cognitive demands associated with prosthetic use, balance and gait disturbances, and brain adaptations [9, 10, 27, 95]. Combined with the fact that dual-tasks represent daily activities, the recommendation is to include dual-task paradigms in the evaluation process of prostheses [95].

The design, development and evaluation of prosthetic devices is an iterative process requiring high cross-disciplinary collaboration between multiple research branches. This review reveals that the current emphasis in prosthetic evaluation has been placed on comparing ankle–foot prostheses without long-term evaluation. Since none of the included studies investigated the long-term benefits of comparing different ankle–foot prostheses, we, for example, cannot make any substantiated statements about the association between the onset of secondary injuries and the use of different types of prostheses solely based on studies conducted at a single point in time. Furthermore, it should be emphasized that the included studies mainly involved people with a transtibial amputation. In contrast, only six of the included studies included people with a transfemoral amputation, limiting the results’ generalisability within the prosthetic population [59, 60, 73–76]. Also, the majority of the studies (94%) are based upon biomechanical and physiological findings during the performance of walking tasks, except for 2, which used performance and subjective measures [63, 86]. Another concern relates to the overall high risk of bias. The high risk of bias can be attributed to the lack of randomisation, the inability to blind participants to the prosthetic condition and the lack of reporting protocol deviations. Specifically, the lack of randomisation and inability to blind participants are essentially inherent to prosthetic research. Taken all of the aforementioned elements into account, heterogeneity of the outcome measures combined with small sample sizes, limited familiarisation time, and the high risk of bias of the included studies do not allow robust conclusions to be made. Therefore, the recommendation is to perform adequate sampled studies with a limited number of outcome measures and ample familiarisation time evaluating a prosthetic device during daily activities. Secondly, the recommendation is shifting the emphasis towards the psychosocial dimension of quality of life through questionnaires finding a suitable poise between objective and subjective measures to obtain a thorough insight into the benefits of prosthetic devices. A recent review provides an overview of psychometric properties of functional, ambulatory, and quality of life instruments to be used in people with a lower limb amputation [43]. At last, we advise conducting prospective studies assessing the benefits of passive, quasi-passive and active prostheses in the longer term similar to those already conducted comparing prosthetic knees or those investigating quality of life after an amputation without comparing prosthetic devices [14, 30, 98–101].

## Conclusion

This review evaluated the differences in the quality of life between passive, quasi-passive and active prostheses for people with a lower limb amputation using biomechanical, physiological, performance and subjective measures. Compared to passive ankle–foot prostheses, quasi-passive and active prostheses improve quality of life. Although short-term therapeutic benefits have been established favouring more advanced prostheses, outcome measures’ discrepancies prevail, the brain’s influence on prosthetic functioning is insufficiently studied, and the long-term benefits remain unknown. Investigating these aspects may improve the quality of life of people with a lower limb amputation.

## Data Availability

All data generated or analysed during this study are included in this published article.
